# Adipocytokine Regulation and Antiangiogenic Activity Underlie the Molecular Mechanisms of Therapeutic Effects of *Phyllanthus niruri* against Non-Alcoholic Fatty Liver Disease

**DOI:** 10.3390/nu10081057

**Published:** 2018-08-09

**Authors:** Raghdaa Hamdan Al Zarzour, Mohammed A. Alshawsh, Muhammad Asif, Majed Ahmed Al-Mansoub, Zahurin Mohamed, Mariam Ahmad, Amin Malik Shah Abdul Majid, Mohd. Zaini Asmawi, Gurjeet Kaur, Dhamraa Waleed Al-dualimi, Mun Fei Yam

**Affiliations:** 1 Discipline of Pharmacology, School of Pharmaceutical Sciences, Universiti Sains Malaysia, Minden 11800, Penang, Malaysia; asif_pharmacist45@yahoo.com (M.A.); majed@usm.my (M.A.A.-M.); aminmalikshah@usm.my (A.M.S.A.M.); amzaini@usm.my (M.Z.A.); dhamora@yahoo.com (D.W.A.-d.); yammunfei@yahoo.com (M.F.Y.); 2 Department of Pharmacology, Faculty of Medicine, University of Malaya, Kuala Lumpur 50603, Malaysia; alshaweshmam@um.edu.my (M.A.A.); zahurin@um.edu.my (Z.M.); 3 Faculty of Pharmaceutical Sciences, Government College University, Faisalabad 38000, Pakistan; 4 Institute for Research in Molecular Medicine (INFORMM), Universiti Sains Malaysia, Minden 11800, Penang, Malaysia; gurjeet@usm.my

**Keywords:** non-alcoholic fatty liver disease (NAFLD), adipose tissues, *Phyllanthus niruri*, adipocytokines, gene expression, antiangiogenic effect

## Abstract

The growth of adipose tissues is considered angiogenesis-dependent during non-alcoholic fatty liver disease (NAFLD). We have recently reported that our standardized 50% methanolic extract (ME) of *Phyllanthus niruri* (50% ME of *P. niruri*) has alleviated NAFLD in Sprague–Dawley rats. This study aimed to assess the molecular mechanisms of action, and to further evaluate the antiangiogenic effect of this extract. NAFLD was induced by eight weeks of high-fat diet, and treatment was applied for four weeks. Antiangiogenic activity was assessed by aortic ring assay and by in vitro tests. Our findings demonstrated that the therapeutic effects of 50% ME among NAFLD rats, were associated with a significant increase in serum adiponectin, reduction in the serum levels of RBP4, vaspin, progranulin, TNF-α, IL-6, and significant downregulation of the hepatic gene expression of PPARγ, SLC10A2, and Collα1. Concomitantly, 50% ME of *P. niruri* has exhibited a potent antiangiogenic activity on ring assay, cell migration, vascular endothelial growth factor (VEGF), and tube formation, without any cytotoxic effect. Together, our findings revealed that the protective effects of *P. niruri* against NAFLD might be attributed to its antiangiogenic effect, as well as to the regulation of adipocytokines and reducing the expression of adipogenic genes.

## 1. Introduction

Non-alcoholic fatty liver disease (NAFLD) is an emerging metabolic disorder that encompasses a wide range of liver injuries, extending from simple steatosis to non-alcoholic steatohepatitis (NASH) which can develop to fibrosis, cirrhosis, and even hepatocellular carcinoma (HCC) [1].

Liver is an essential organ for normal lipid homeostasis [2]. Free fatty acids (FFAs) are released into the bloodstream through lipolysis process in adipose tissues, and the hepatocytes are responsible for their uptake by fatty acid carriers [3]. In hepatocytes, FFAs are found in various pathways. For instance, in fasting state, they are oxidized in the mitochondria and peroxisomes to produce energy, while in fed state, they might be re-esterified into triglycerides. Additionally, glucose is converted to FFAs in the liver by means of de novo lipogenesis (DNL) which produces triglycerides [4].

NAFLD is frequently correlated with obesity and insulin resistance, at the level of adipose tissue and liver. The adipose tissue in this condition becomes insensitive to the antilipolytic influence of insulin, which leads to an increase in the amount of released fatty acids. The β-oxidation of FFAs in the liver is declined [5]. Moreover, elevated levels of insulin, along with increased fat intake, promote the synthesis of hepatic triglycerides through de novo lipogenesis process, leading to abnormal storage of triglycerides in the liver. More specifically, the imbalance between import and export of FFAs induces a hepatic triglycerides accumulation which presents benign steatosis. However, adipocytokines and oxidative stress are proposed to cause hepatocyte damage through the inflammatory response. NAFLD progresses from steatosis to non-alcoholic steatohepatitis (NASH), and causes fibrosis and malignancy stages [6].

The de novo lipogenesis and uptake of FFA are mainly controlled by SREBP-1c gene (sterol regulatory element binding protein-1c) and carbohydrate responsive element-binding protein (ChREBP). Both SREBP-1c and ChREBP activate the expression of key biosynthetic enzymes specific for fatty acid synthesis, such as acetyl-CoA carboxylase (ACC), fatty acid synthase (FAS), and stearoyl-CoA desaturase 1 (SCD1) [7]. In addition, PPARα activates β-oxidation by upregulating key genes, such as CPT1 (carnitine palmitoyltransferase) and suppressing SREBP-1c [8].

Many recent studies proposed that dysfunction in adipose tissue has been linked to the pathogenesis and progress of NAFLD. The lipotoxicity of accumulated fats in the liver triggers secretion of pro-inflammatory adipocytokines [9]. Moreover, recent interesting findings suggest that angiogenesis is a unique feature of early stages of NAFLD, showing a positive correlation between fibrosis stage and angiogenic activity [10].

Taking into consideration the vital role of specific genes and adipocytokines on hepatic steatosis, fibrosis, and inflammation, researchers worldwide have strengthened their efforts to study the mechanistic pathways underlying the effects of therapeutic agents on adipocytokines and genes which are thought to contribute in NAFLD progression.

*Phyllanthus niruri (P. niruri*) belongs to the Euphorbiaceae family, and grows in tropical and subtropical regions of the world. Due to its wealthy medicinal properties, it has been used extensively for more than 2000 years by different nations, such as Malaysia, and in Unani medicine and Indian Ayurvedic therapeutics for stimulating liver in treatment of jaundice, asthma, and bronchitis [11]. *P. niruri* extract has shown potent hepatoprotective and antioxidant effects, and antihyperlipidemic activities [12,13].

Despite our earlier encouraging data, which confirmed that a standardized 50% methanolic extract (50% ME) of *P. niruri* inhibited the progression of NAFLD in Sprague–Dawley (SD) rats, reduced NAFLD activity score (NAS), prevented fibrosis, and improved serum levels of liver enzymes, glucose, insulin, insulin resistance, lipid profile, serum free fatty acids (FFAs), and hepatic contents of total cholesterol and triglyceride [14], to date, the pathways underling the molecular mechanism of action have not yet been elucidated. Therefore, this study aimed to assess the mechanism of action and the role of adipocytokines underlying the therapeutic effect of 50% ME of *P. niruri* against NAFLD, as well as to study the expression of some genes by which *P. niruri* extract have been expected to exert its activities. We also aimed to further investigate the antiangiogenic activity of 50% ME of *P. niruri* in vitro.

## 2. Material and Methods

### 2.1. Extract Preparation 

The whole plant parts of *P. niruri* were collected and a voucher specimen (No. 11474) was kept in the Herbarium Unit at School of Biological Sciences, Universiti Sains Malaysia. The plant was rinsed with clean water, and dried, then was ground into powder. The dry powder was macerated by continuous stirring with 50% methanol/water at 40 °C for 48 h, and then evaporated under reduced pressure and completely dried. The dried extract was stored at 4 °C until further use.

### 2.2. Animals

In total, 24 male Sprague–Dawley (SD) rats aged (10 weeks old) were provided by the Animal Research and Service Centre, Universiti Sains Malaysia. All experimental protocols and methods were performed in accordance with the relevant guidelines and regulations of the Experimental and Animal Ethics Committee of the School of Pharmaceutical Sciences, Universiti Sains Malaysia (protocol No. 2013/(90) (546)). Rats were fed with a high-fat diet (HFD) for 8 weeks to induce NAFLD, as previously described [14]. Animals were randomly divided into four groups (*n* = 6). Group (1), the normal control group, was fed a normal diet for 8 weeks, while the other three groups (2, 3, and 4) were fed with HFD for 8 weeks. Treatment was applied from week 5. Group (1) was treated with distilled water (10 mL/kg body weight). Group 2, the negative control group, was treated with distilled water (10 mL/kg body weight); group 3, the positive control group, was treated with metformin (500 mg/kg body weight), and group 4 received the treatment of 50% ME of *P. niruri* (1000 mg/kg body weight).

At the end of week 8, rats were fasted overnight and anesthetized. Blood was collected via cardiac puncture, and centrifuged to get the serum, which was stored at −80 °C until further use. Liver samples were removed from the largest hepatic lobe, washed with a chilled 0.9% NaCl solution, dried, and weighted. Then, liver samples were divided into two parts: one part was fixed in formaldehyde 10% (*v*/*v*) for histological analysis, while the other part was immediately kept in RNA later solution (QIAGEN, Hilden, Germany), and stored at 4 °C overnight to prevent RNA enzymatic degradation. Then, all samples were placed in the freezer at −80 °C until further use for gene expression analysis. Serum samples were used to determine hepatic liver enzymes.

### 2.3. Histological Examination

Liver sections were embedded in paraffin and stained with hematoxylin and eosin (H&E) stain to evaluate steatosis and inflammation, and also stained with Masson’s trichrome for confirmation of fibrosis.

### 2.4. Effects of P. niruri on Serum Adipocytokines

To evaluate the effect of 50% ME of *P. niruri* and metformin treatment on serum adipocytokines in NAFLD-induced rats, serum adiponectin, RBP4, and progranulin were measured by rat ELISA kits (AdipoGen, Liestal, Switzerland) according to the manufacturer’s protocols. Serum TNFα, IL-6, and vaspin concentrations were determined by sandwich enzyme immunoassay technique (ELISA) (CusaBio, Wuhan, China).

### 2.5. Gene Expression Assessment by Real Time PCR

Liver specimens collected from sacrificed rats were kept immediately in the preservative RNA later solution. (Qiagen, Hilden, Germany) at 4 °C overnight, and then were stored in −80 °C until used. Approximately 30 mg of the frozen liver tissues were mixed with lysis buffer and homogenized. Total RNA was extracted using RNeasy plus Mini Kit (Qiagen, Hilden, Germany) according to the manufacturer’s protocol.

RNA purity was measured spectrophotometrically via a NanoDrop ND-2000c Spectrophotometer (Thermo Scientific, Wilmington, DE, USA), and the ratio of the readings at 260 nm and 280 nm (A260/A280) was estimated. RNA integrity was quantified by using Agilent 2100 bioanalyzer (Agilent Technologies, Santa Clara, CA, USA).

RNA was reverse-transcribed to cDNA using Transcriptor First Strand cDNA Synthesis Kit (Roche Applied Science, Mannheim, Germany), according to the manufacturer’s protocol. Concentration of the resulting cDNA was measured spectrophotometrically, and was amplified using TaqMan rat assay genes from Applied Biosystems (Foster, CA, USA). The amplification reaction was performed using StepOnePlus Real-Time PCR System (Applied Biosystems, Foster, CA, USA).

Quantitative Real-Time PCR (qRT-PCR) was carried out for the following genes: peroxisomal proliferator-activated receptor-γ (P PARγ) (NM_001145366.1), solute carrier family 10 (sodium/bile acid cotransporter) member 2) (SLC10A2) (NM_001270862.1), complement factor D (adipsin) (CFD) (NM_001077642.1), patatin-Like phospholipase domain containing 2 (PNPLA2) (NM_001108509.2), collagen alpha 1 (Coll α1) (NM_053304.1), and glyceraldehyde 3-phosphate dehydrogenase (GAPDH) (NM_017008.4) and hypoxanthine phosphoribosyl transferase (Hprt1) (NM_012583.2) were used as housekeeping genes (Table 1). All measurements were done in triplicate.

The amount of target transcript in each sample was normalized to GAPDH and HPRT-1, and the averages of the obtained threshold cycle (Ct) values were processed for further calculations according to the comparative Ct method. Fold change was determined according to the arithmetic formula 2^−ΔΔCt^ method [15] and ΔΔCT was calculated via the following formula:

ΔΔCT = ΔCT (sample) − ΔCT (normal)(1)

ΔCT represents the difference in Ct value between the targeted gene and the housekeeping genes.

### 2.6. Ex Vivo Aortic Ring Assay

A standard method was used to screen the antiangiogenic potency of 50% ME of *P. niruri* whole plant extract [16]. Photos were taken for aortic rings and examined microscopically under an inverted light microscope (4× magnification). The length of blood vessels in each group was measured using ImageJ analysis software. Percent inhibition of blood vessel outgrowth in *P. niruri* treatment groups was calculated by comparing length of blood vessels in control (0.5% dimethyl sulfoxide (DMSO)) and treated rings (*P. niruri*).

### 2.7. MTT Cell Proliferation Assay

The antiproliferative effects of 50% ME of *P. niruri* against somatic endothelial cells (EA.hy926) were studied using MTT cell viability assay following a standard method [17,18]. Absorbance of dissolved formazan crystals was measured at 570 nm using 620 nm as reference wavelength. Media with 0.5% DMSO and betulinic acid (10 µg/mL) were used as negative and positive controls, respectively. Values shown are mean ± SD of three independent experiments.

The IC_50_ value of *P. niruri* was determined from the logarithmic regression equation by plotting a graph between different concentrations and mean percent inhibition of cell proliferation. After determination of IC_50_, all the other experiments were carried out using three different concentrations i.e., IC_25_, IC_50_, and IC_90_ of *P. niruri*. Values shown are mean ± SD of three independent experiments.

### 2.8. Migration Assay

The scratch assay was used to study the anti-migratory effects of 50% ME of *P. niruri* against EA.hy926 cells [19,20]. Media containing 0.5% DMSO and betulinic acid (10 µg/mL) were used as negative and positive controls, respectively [20]. The photographs of each well at six different points were taken at different time points (0T and 12T), and the area of the cell-free zone was measured using an ImageJ software plugin (units = µm). Results are presented as mean percentage inhibition of cell migration ± SD of three independent experiments (*n* = 3).

### 2.9. Tube Formation Assay 

The ability of 50% ME of *P. niruri* extract to arrest the differentiation of EA.hy926 cells into tube-like structures on Matrigel matrix was evaluated using a reported method [16]. DMSO and betulinic acid (10 µg/mL) were used as negative and positive controls, respectively. Images were analyzed using WinTube imaging solution of WIMASIS software (WIMASIS Image Analysis, Munich, Germany). Percent inhibition of tube formation was calculated by comparing the total area covered in 0.5% DMSO (control) and *P. niruri* treated cells [21]. The results are expressed as the mean percentage of inhibition ± SD (*n* = 3).

### 2.10. Determination of Vascular Endothelial Growth Factor (VEGF) Concentration in EA.hy926 Cell Lysates

Human VEGF Duoset ELISA kit (R&D systems, Minneapolis, MN, USA) was used to determine the effect of 50% ME of *P. niruri* on the levels of VEGF secreted from human colon cancer HCT 116 cells. Media with 0.5% DMSO was used as negative control. The *R*^2^ value obtained from linear regression equation of VEGF standard calibration curve was used to calculate the concentration of VEGF in the 0.5% DMSO-treated and *P. niruri*-treated cells, and the percent inhibition of VEGF secretion in *P. niruri*-treated cells was calculated using a reported equation. The values presented are in mean ± SD (*n* = 3).

(2) $$\%\;\mathrm{Inhibtion}\;\mathrm{of}\;\mathrm{VEGF}\;\sec\mathrm{rection}=\left[1-\left(\frac{\mathrm{VEGF}\;\mathrm{sample}}{\mathrm{VEGF}\;\mathrm{control}}\right)\times 100\right]$$

### 2.11. Statistical Analysis 

The obtained data were expressed as means ± SEM. Statistical significance was analyzed using version 20 of the Statistical Package of Social Sciences (SPSS) statistical program (IBM Corp., Armonk, NY, USA). Real-time PCR results were analyzed using GenEX5 program (http://www.multid.se) [22], and fold differences were calculated. Differences between groups were assessed using the one-way analysis of variance (ANOVA), followed by Dunnett’s test as a post hoc test; and they were considered significant at *p* < 0.05.

## 3. Results

### 3.1. Effects of P. niruri on Liver Histopathology

**Figure 1:**
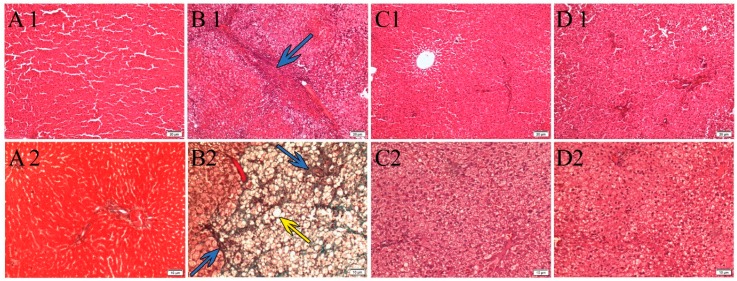
Photographs of the liver tissues stained by H&E (**A1**,**B1**,**C1**,**D1**) and by Masson’s trichrome staining (**A2**,**B2**,**C2**,**D2**), all under magnification (100×), showing the effect of metformin and *P. niruri* treatment on the liver histology of non-alcoholic fatty liver disease (NAFLD) rats. (**A1**,**A2**) showing normal architecture of liver tissue without any sign of steatosis in the normal control group which received normal diet; (**B1**,**B2**) high-fat diet HFD group which represents untreated NAFLD rats, showing micro and macro steatosis with fibrosis; (**C1**,**C2**) showing improvement in liver histology of NAFLD rats which were treated with metformin, and (**D1**,**D2**) showing improvement in liver histology of NAFLD rats which were treated with 50% methanolic extract (ME) of *P. niruri.* Yellow arrow refers to hepatic steatosis, while blue arrow refers to hepatic fibrosis.

Histological changes in liver sections from each group are presented in Figure 1. Hematoxylin and eosin (H&E) staining showed that the liver tissue of the normal control group had no signs of inflammation, steatosis, or fibrosis. On the other hand, severe hepatic micro- and macrovesicles of hepatic steatosis were identified in the liver of HFD group. The hepatocytes in this group were significantly greater in size, compared to normal control group which was fed with normal diet. Fibrosis and hepatic damage were observed only in HFD group, which was confirmed by Masson’s trichrome staining (Figure 1). Conversely, treatment with *P. niruri* extract and metformin clearly reduced steatosis and prevented fibrosis.

### 3.2. Effects of 50% ME of P. niruri on Serum Adipocytokines

**Figure 2:**
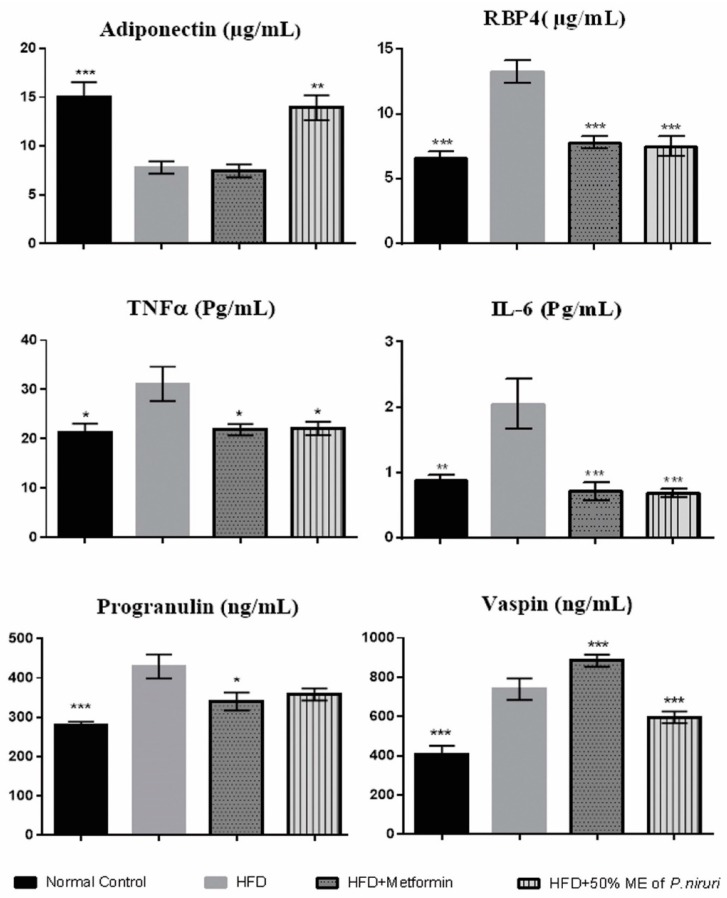
Effects of *P. niruri* on adiponectin, RBP4, TNFα, IL-6, progranulin, and vaspin in the different groups. * *p* ˂ 0.05 vs. HFD group, ** *p* ˂ 0.01 vs. HFD group, *** *p* < 0.001. Data expressed as mean ± SEM.

As shown in (Figure 2), the consumption of HFD caused a significant (*p* < 0.01) reduction in serum adiponectin levels in HFD group (7.80 ± 0.63 µg/mL) compared with normal group (15.05 ± 1.49 µg/mL). However, the concentration of serum adiponectin was significantly increased (*p* < 0.01) after the treatment with *P. niruri* (13.93 ± 1.28 µg/mL) compared with HFD group, while serum adiponectin level was not improved in rats treated with metformin. On the other hand, serum levels of both TNFα and IL-6 were significantly (*p* < 0.05) increased in HFD group compared with normal diet group, while the treatment with *P. niruri* and metformin significantly inhibited this elevation in circulating levels of serum TNFα (*p* < 0.05) and IL-6 (*p* < 0.01) compared with HFD group. Moreover, there was a considerable increase in the serum levels of RBP4 (*p* < 0.01) in the high-fat diet group (13.28 ± 0.86 µg/mL) compared with normal diet group (6.61 ± 0.49 µg/mL). However, this trend was significantly (*p* < 0.01) reversed in rats treated with *P. niruri* (7.50 ± 0.77 µg/mL) and metformin (7.80 ± 0.49 µg/mL) groups.

Serum concentration of progranulin was significantly (*p* < 0.01) higher in the HFD group (429.43 ± 30.38 ng/mL) and it was non-significantly (*p* = 0.065) lowered (358.15 ± 15.21 ng/mL) after the treatment with *P. niruri*, and significantly (*p* < 0.05) reduced in rats treated with metformin (340.37 ± 22.68 ng/mL).

The concentration of serum vaspin was significantly (*p* < 0.01) elevated in the HFD group (739.11 ± 22.23 ng/mL) compared with the normal control group (407.65 ± 17.49 ng/mL). However, this value was significantly (*p* < 0.01) decreased in the group treated with *P. niruri* (595.72 ± 12.19 ng/mL) compared with the HFD group, however, surprisingly, this value was even significantly higher (*p* < 0.01) in metformin-treated group (884.02 ± 12.35 ng/mL).

### 3.3. Gene Expression

#### 3.3.1. RNA Purity and Integrity

Spectrophotometric measurements using a NanoDrop for all samples ranged from 1.99–2.11, which indicates good quality of RNA. In addition, the RNA integrity number (RIN) was checked via Agilent 2100 Bioanalyzer, and demonstrated that RIN ranged between 7 and 10 for all samples, and the apparent ratio of 28S to 18S ribosomal RNA was approximately 2:1 (data not shown), suggesting that our extracted RNA samples were intact and could be used for real-time PCR.

#### 3.3.2. Quantitative Real-Time PCR (qRT-PCR) for Expression Analysis

The expression levels of PPARγ, SLC10A2, CFD, PNPLA2, COL1α1, and the two endogenous reference genes were subsequently validated by qRT-PCR measurements. All genes showed that the efficiency was between 90–110%, and the slope between (−3.1) and (−3.5), which are within the reference criteria to run the qRT-PCR. After data analysis of the Ct values by GenEX5 program and normalizing to the reference genes HPRT-1 and GAPDH, all measured mRNAs showed a different expression level between calibrator group (normal liver tissue) and negative group (HFD group). Findings showed that high-fat diet caused highly significant (*p* < 0.01) upregulation of three target genes (PPARγ, SLC10A2, and Collα1), compared to the calibrator (normal rats), while causing insignificant downregulation in PNPLA2 gene (0.45 ± 0.07) and upregulation in CFD gene (2.01 ± 0.14).

**Figure 3:**
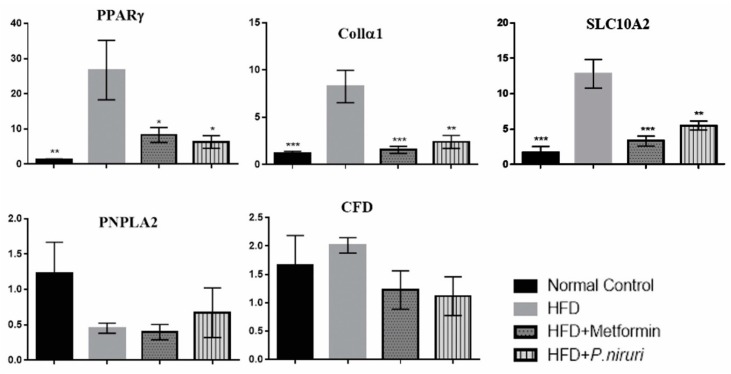
Real-time PCR analysis shows the relative normalized gene expression of peroxisomal proliferator-activated receptor-γ (PPARγ), collagen type alpha 1 (Collα1) and sodium/bile acid cotransporter member 2 (SLC10A2) between all experimental groups. All values are expressed as mean fold changes of mRNA ± SEM, * *p* ˂ 0.05 vs. HFD group, ** *p* ˂ 0.01 vs. HFD group, *** *p* ˂ 0.001 vs. HFD group.

The expression of PPARγ, SLC10A2, and Collα1 genes in the HFD group were upregulated by 26.76 ± 8.46-, 12.82 ± 2.03-, and 8.25 ± 1.71-fold, respectively, compared to the calibrator (normal rats), whereas the fold change values in *P. niruri*-treated group were significantly downregulated by 20.5 ± 8.6 (*p* < 0.05)-, 7.3 ± 2.1 (*p* < 0.01)-, and 5.9 ± 1.8 (*p* < 0.01)-fold, respectively, compared with the expression in HFD group. Additionally, the expression of these genes in metformin-treated group were also significantly downregulated by 18.5 ± 8.7 (*p* < 0.05)-, 9.5 ± 2.2 (*p* < 0.01)-, and 6.7 ± 1.8 (*p* < 0.01)-fold, respectively, in comparison to HFD group (Figure 3).

### 3.4. Antiangiogenic Activity of 50% ME of P. niruri

#### 3.4.1. *P. niruri* Whole Plant Extract Blocks the Growth of Microvessels

**Figure 4:**
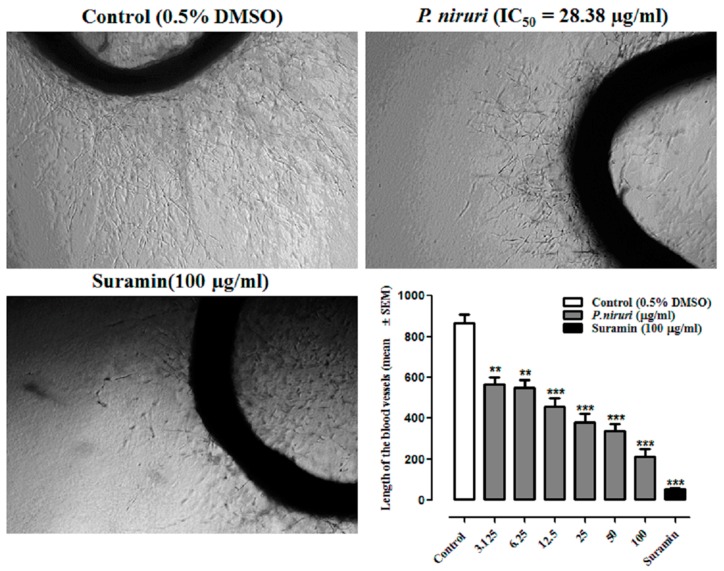
Photomicrographic represents the anti-neovascularization effects of *P. niruri* whole plant extract in rat aortic ring assay. Photos were taken at 4× magnification (scale bar 1000 µm). Where *p* ** < 0.01 and *** *p* < 0.001 vs. control Suramin; DMSCO: dimethyl sulfoxide.

Significant inhibition of blood vessel outgrowth was observed in thoracic rat aortic rings treated with different concentrations of *P. niruri* extract (Figure 4). Percent inhibition of blood vessel outgrowth in aortic rings treated with 6, 12, 25, 50, and 100 µg/mL of *P. niruri* was 36%, 45%, 53%, 66%, and 78%, respectivley. The IC_50_ value of *P. niruri* whole extract, as calculated from the linear regression equation (y = 0.6556x + 30.405, *R*^2^ = 0.9223) of dose-response curve, was 28.38 ± 0.82 µg/mL. Suramin, at the concentration of 100 µg/mL, caused 90% inhibition of blood vessel outgrowth.

#### 3.4.2. *P. niruri* Extract Induces No Toxicity towards Normal Endothelial EA.hy926 Cells

**Figure 5:**
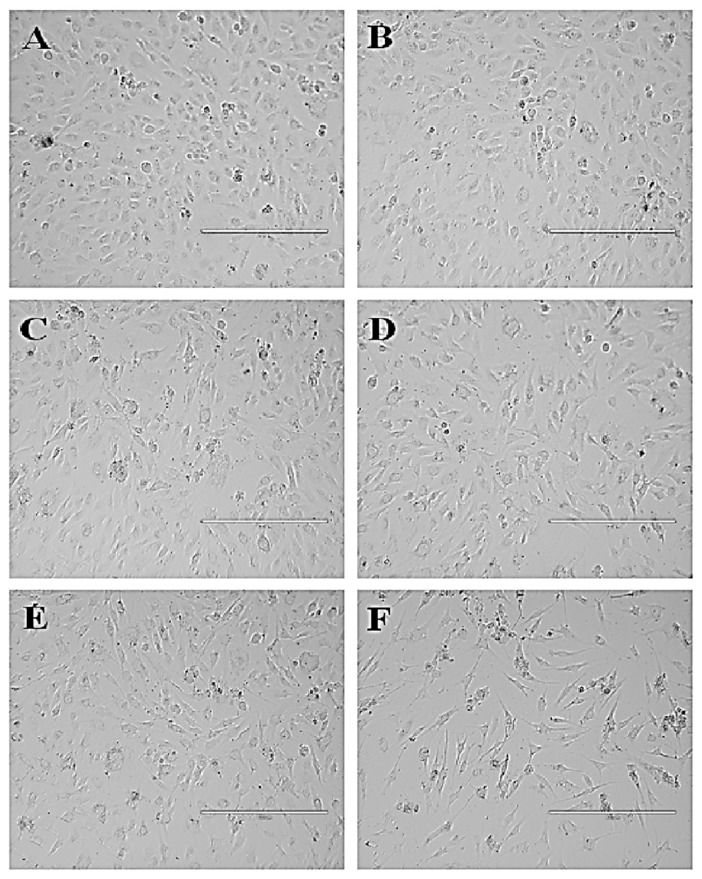
Photomicrographic represents effect of 50% ME of *P. niruri* treatment on EA.hy926 cell viability. Where (**A**) = 0.5% DMSO (control), (**B**) = 12 µg/mL *P. niruri*, (**C**) = 25 µg/mL *P. niruri*, (**D**) = 50 µg/mL *P. niruri*, (**E**) = 100 µg/mL *P. niruri*, and (**F**) = 10 µg/mL betulinic acid (+ve control). Photos were taken at 10× magnification (scale bar 400 µm).

The MTT cell viability assay revealed that 50% ME of *P. niruri* whole plant extract did not induce cytotoxicity towards EA.hy926 cells at the tested concentrations (100–3.125 µg/mL). The IC_50_ of 50% ME of *P. niruri* extract was calculated to be 136.26 ± 7.76 µg/mL. Based on the IC_50_ value of 50% ME of *P. niruri* towards EA.hy926 cells, all further in vitro assays were conducted using three different concentrations (IC_25_ = 68 µg/mL, IC_50_ 136 µg/mL and IC_90_ = 244 µg/mL). Figure 5 shows the cytotoxic effect of 50% ME of *P. niruri* against EA.hy926 cells. No apparent changes in morphology of EA.hy926 cells were observed in *P. niruri* extract treatment groups.

#### 3.4.3. *P. niruri* Extract Inhibits Migration of EA.hy926 Cells

**Figure 6:**
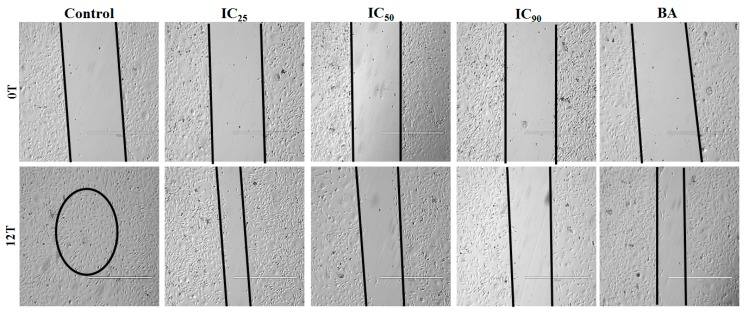
The anti-migratory effects of 50% ME of *P. niruri* against EA.hy926 cells. Photos were taken at 4× magnification (scale bar 1000 µm), BA: betulinic acid.

Dose-dependent reduction in migration potency of EA.hy926 cells across the artificially created wound was observed in 50% ME of *P. niruri* treatment groups. Percent inhibition of cell migration after 12 h of treatment with IC_25_, IC_50_, and IC_90_ concentrations of *P. niruri* extract was 73.50 ± 4.63%, 85.26 ± 12.21%, and 93.50 ± 6.04%, respectively. Figure 6 shows photomicrographs of anti-migratory effects of *P. niruri* extract towards EA.hy926 cells.

#### 3.4.4. *P. niruri* Extract Inhibits the Differentiation of EA.hy926 Cells into Tube-Like Structures

**Figure 7:**
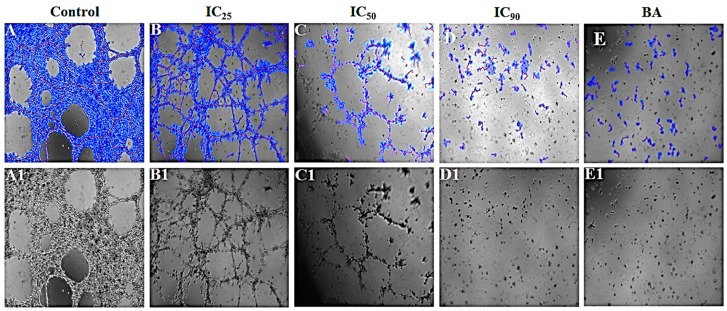
Photomicrographic representations of the effect of 50% ME of *P. niruri* whole extract on EA.hy926 cell tube formation. (**A**–**E**) represent images analyzed using Wimasis Image Analysis software (Wimasis GmbH, Munich, Germany), while (**A1**–**E1**) represent raw images. BA: betulinic acid.

Dose-dependent inhibition of differentiation of EA.hy926 cells into tube-like structures was observed in 50% ME of *P. niruri* treatment groups. *P. niruri* extract caused significant reduction in the percentage of area occupied by the formed tubes, total length of tubes, number of loops formed, and number of branches, separately. Pecentage inhibition of tube formation at IC_25_, IC_50_, and IC_90_ of *P. niruri* extract was 35.58 ± 16.30%, 71.07 ± 4.78% and 87.58 ± 1.47% respectively, while percent inhibition of tube formation in betulinic acid treated group was 77.07 ± 9.64%. Table 2 summarizes the effect of *P. niruri* extract against different parameters of tube formation. Figure 7 depicts the antiangiogenic effects of *P. niruri* in tube formation assay.

#### 3.4.5. *P. niruri* Extract Inhibits the Release of VEGF Secretion from HCT 116 Cells

**Figure 8:**
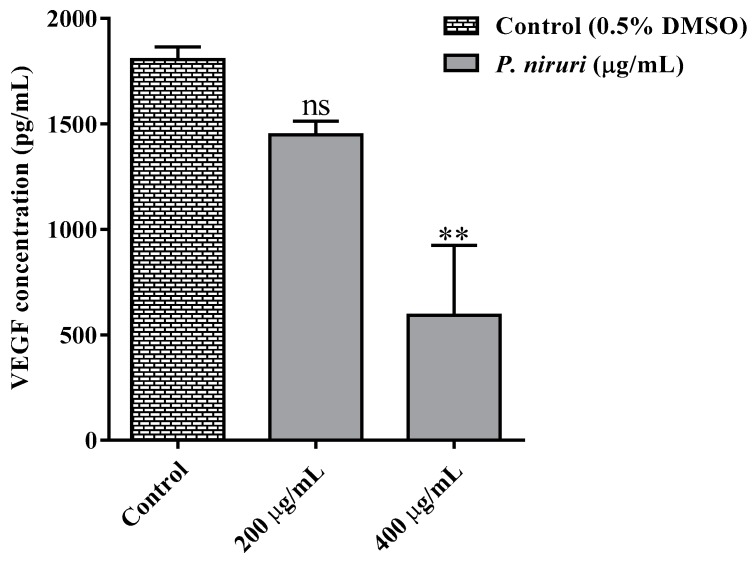
Effects of 50%ME of *P. niruri* on vascular endothelial growth factor (VEGF) concentration compared with the control (DMSO) according to ELISA examination kit. All values are expressed as mean ± SEM, ** *p* ˂ 0.01 vs. the control (DMSO).

As shown in Figure 8, treatment with 200 and 400 µg/mL of 50% ME of *P. niruri* extract resulted in dose-dependent inhibition of VEGF release from HCT 116 cells. Percent inhibition of VEGF released in *P. niruri* extract treatment groups (200 and 400 µg/mL) was 44.27% and 67.11%, respectively. 

## 4. Discussion

**Figure 9:**
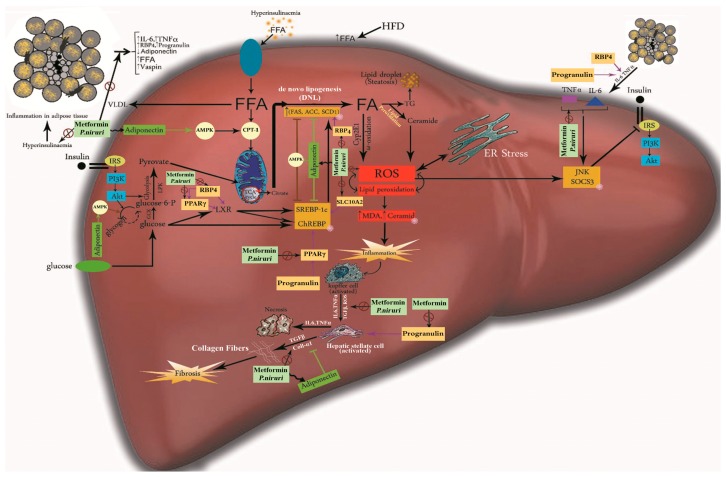
Illustration summarizes the proposed underlying mechanism of action of metformin and *P. niruri* against NAFLD.

Mitochondrial dysfunction and the overload of fats, along with insulin resistance, are considered the main pathological factors of NAFLD development. The accumulated fat results in endoplasmic reticulum (ER) stress, with a rise in the levels of reactive oxygen species (ROS) inside the hepatocytes in the liver [23]. This oxidative stress, along with other factors, promote the inflammatory reactions that might progress to fibrosis and/or hepatic carcinoma [24] (Figure 9).

Many recent studies proposed that adipose visceral tissue is an essential metabolic and inflammatory organ, and secretes an array of hormones (adipocytokines) which are transported directly to the liver though its portal vein [25].

According to the current data, oral feeding with the HFD has resulted in obvious dysfunction in adipose tissue, shown by the significant reduction in the serum levels of adiponectin, with significant increase in TNF-α, IL-6, RBP4, progranulin, and vaspin levels compared with normal diet group. However, ameliorating these pathological changes by 50% ME of *P.niruri* treatment which also reversed steatosis as well as liver fibrosis, is a clear indicator of the therapeutic effects of *P. niruri* on both adipose tissues and liver.

Adiponectin is involved in increasing insulin sensitivity and fatty acid oxidation through the activation of PPARα and AMPK pathway. It prevents cellular proliferation and cell death by enhancing ceramide catabolism [26]. Adiponectin has also anti-inflammatory effects by the inhibition of TNF-α production and blocking the activity of nuclear factor-kB (NF-kB). It inhibits fibrosis by suppressing proliferation and migration of the hepatic stellate cells (HSC) and attenuating gene expression of transforming growth factor beta 1 (TGF-β1) [27]. In this study, the hepatic damage in untreated NAFLD rats was associated with a remarkable decrease in the circulating levels of adiponectin, while the increase of these levels in rats treated with *P. niruri* improved insulin sensitivity and attenuated liver inflammation. Adiponectin, with its insulin-sensitizing and anti-inflammatory effects in NAFLD model, was one of the main factors to prevent fibrosis by reducing the concentrations of TNF-α and IL-6, and consequently, inhibit the inflammatory response. We propose that elevated circulating levels of adiponectin are likely to promote the AMPK signaling pathway in hepatocytes, suppress de novo lipogenesis by inhibiting SREBP1c, ChREBP, ACC, and FAS, as well as suppressing ER stress by activating CPT-1 to improve the β-oxidation of FFAs, leading to significant increase in insulin sensitivity with protective effects against hepatic steatosis and insulin resistance. These results are in agreement with the reported effects of adiponectin in NAFLD [28]. Moreover, our results of the efficacy of metformin through increasing adiponectin serum concentration in NAFLD is in accordance with other previous studies [29].

Tumor necrosis factor-α (TNF-α) enhances lipolysis and fatty acid release from adipocytes, blocks insulin signals, and stimulates hepatic lipogenesis through activating c-Jun N-terminal kinase (JNK1), which also inhibits insulin signals [26]. TNF-α promotes inflammation through the NF-kB pathway. Even more, it promotes the expression of the suppressor of cytokine signal 3 (SOCS3), which inhibits insulin signal by activating SREBP-1c [30]. Interleukin 6 (IL-6) is another inflammatory cytokine, strongly correlated with insulin resistance in the liver through JNK and via increasing the expression of SOCS3 [31]. Both TNF-α and IL-6 are involved in the conversion of HSCs into myofibroblasts that contribute to liver fibrosis [32]. In the present study, both TNF- α and IL-6 were significantly increased in the serum of HFD rats, while treatment with *P. niruri* and metformin decreased the serum levels of both TNF-α and IL-6 in HFD-fed rats. This result is in agreement with previous experimental work which has reported the anti-inflammatory activity of *P. niruri* [33] and metformin [34].

Retinol binding protein 4 (RBP4) is one of the transport proteins for vitamin A, and its higher levels are associated with insulin resistance. It was demonstrated that RBP4 stimulates lipogenesis in hepatocytes by activating the expression and translocation of SREBP1, inducing the expression of downstream genes, such as FAS and ACC1, which all mediate the de novo lipogenesis in the hepatocytes and block insulin signals [35]. This lipogenic effects of RBP4 are intermediated by PPARγ and liver X receptor (LXR) [36]. Moreover, according to a recent study, RBP4 induces hepatic steatosis because it activates primary inflammatory effects in adipose tissue and stimulates proinflammatory cytokines, such as TNF-α [37], while the suppression of RBP4 prevented NAFLD in mice models [38].

In the current work, findings showed that serum levels of RBP4 were decreased in groups treated with metformin and *P. niruri*. These data confirmed the reported effect of metformin to reduce RBP4 as an indicator for an improvement of insulin sensitivity to prevent steatosis [39]. However, to the best of our knowledge, this is the first report about the potential effect of *P. niruri* to reduce RBP4 as an effective pathway to prevent steatosis and reduce insulin resistance.

Progranulin is another adipokine linked with insulin resistance and obesity. It is usually not expressed in normal hepatocytes, but is highly expressed in the liver of NASH mouse model induced by a high-fat diet [40]. Progranulin is positively correlated with insulin resistance, HOMA-IR, and circulating PPARγ [41]. Recent data reported that progranulin levels have been raised in hepatic stellate cells and linked with liver fibrosis [42]. It was also found that serum progranulin contributes to developing insulin resistance through increasing IL-6, which in turn, impairs insulin signaling by stimulating SOCS3 expression [43].

Similarly to what was reported in other pharmacological studies, we have noticed, in our study, that progranulin serum levels were higher in the HFD group, confirming its positive relationship with insulin resistance and HOMA-IR, and its association with hepatic damage and fibrosis that was reported previously [42]. The decrease of progranulin concentration in *P. niruri*-treated rats was significant, while metformin treatment was able to induce considerable reduction in serum adipocytokine concentrations.

Vaspin is one of the most novel recognized adipokines, and it is known as a visceral adipose tissue (VAT)-derived serine protease inhibitor. It was proposed that vaspin serves as an insulin sensitizer with anti-inflammatory effects. Increased circulating concentration of vaspin during insulin resistance in obesity can be explained by its compensatory mechanism, targeting adipose tissues and antagonizing the disrupting effects of unknown proteases in insulin action [44], such as the inhibitory effect on human kallikrein (hK7) that mediates the degradation of insulin in circulation [45].

Findings of our current work demonstrated that circulating vaspin concentration was increased in HFD rats compared with rats fed a normal diet. This result confirmed that elevated vaspin represents a bodily defense against NAFLD [46]. However, only treatment with *P. niruri* could decrease serum vaspin concentrations compared with the HFD group, but they were significantly increased in metformin-treated rats. This may be explained as a compensatory increase, which was reported for vaspin in previous studies [47]. However, different findings, reported in human, showed that metformin has lowered vaspin levels in overweight women, leading to improvement in insulin sensitivity [48]. In agreement with our finding, a study by Gonzales et al. has shown that metformin increased vaspin expression in adipose tissues in rats. It was concluded, in that study, that the effect of metformin on vaspin might be different between humans and rodents [49]. As a result, more experimental work is recommended to reveal the mechanism of metformin involved in changing serum levels of vaspin.

PPARγ is a novel member of the PPAR family which was suggested to be a major regulator in adipose tissue. PPARγ exhibits a great adipogenic action in the pathological state of NAFLD, and it stimulates the metabolism and storage of lipids [50]. It was found that rosiglitazone, a PPARγ agonist, seriously increases hepatic steatosis, due to its elevated hepatic expression of PPARγ [51], while deleting PPARγ in liver cells protected mice against hepatic steatosis induced by high-fat diet [52]. One of the suggested mechanisms in which PPARγ contributes in hepatic steatosis is by enhancing the de novo lipogenesis through stimulating LXR, which in turn activates SREBP-1c, ChREBP, FAS, and ACC [53]. However, PPARγ can be phosphorylated and inactivated by AMPK pathway [54].

Our gene expression results showed that there was significant positive association between the upregulation of PPARγ and the elevated circulating levels of progranulin, vaspin, and RBP4 in the HFD group which represents NAFLD rats. This relationship was also investigated by [55].

Collagen type 1 α1 (Collα1) gene is a tumor-related gene. This human gene has been described to be a vital component of hepatic fibrosis regulated by TGF-β in activated HSCs [56]. It has been found that Collα1 expression is upregulated in NAFLD [57]. On the other hand, it has been reported that this gene is downregulated by adiponectin [58]. The significant increase in the proinflamatory cytokines TNF-α, IL-6, RBP4, and progranulin, has contributed to promoting insulin resistance and triggering the inflammatory response, resulting in activation of HSCs to produce collagen and stimulate liver fibrosis.

SLC10A2 is a member of the sodium/bile salt cotransporter family, which has been identified in human and rats [59,60]. SLC10A2 is critical factor in the enterohepatic circulation of bile salts. It actively mediates intestinal absorption of bile acids, and its functions include removing bile salts from the ileum and facilitate their return back to the liver through portal circulation [61]. This represents an essential route for cholesterol elimination from the body. Interrupting the enterohepatic circulation of the bile acids promotes cholesterol catabolism, and increases hepatic cholesterol synthesis starting from acetate. Recent studies suggested decreasing the hepatic acetate pool in order to affect glucose and triglyceride metabolism by inhibiting SLC10A2. It was found that blocking SLC10A2 lowered serum TG, as well as hepatic production of TG, inhibited SREBP-1c, and normalized insulin concentrations in obese diabetic mice (ob/ob) model. Therefore, SLC10A2 inhibitors seem to be successful agents for attenuating hepatic lipogenesis by blocking SREBP-1c [62] and treatment of NAFLD [62,63]. Accordingly, the overexpression of PPARγ and SLC10A2 in the HFD group might be a vital reason for hepatic steatosis [50,64]. Nevertheless, the reduction in hepatic steatosis and fibrosis in groups treated with *P. niruri* extract and metformin is associated with an effective reduction in the hepatic mRNA of PPARγ, SLC10A2, and Coll α1. These results provide a wealth of evidence about the beneficial effect of targeting these genes in treatment of NAFLD.

A recent study by Gao et al. has proposed that the pathological changes in circulating levels of adipocytokines during NAFLD progression are linked with the expansion of adipose tissues, concomitantly with a release of extracellular vesicles from adipocytes, which is involved in stimulating insulin resistance and augmenting growth factor β expression, inflammation, and fibrosis in the liver [65]. Moreover, it was reported by Kim et al. that this expansion of adipose tissues during NAFLD has been found to be angiogenic dependent. Therefore, it was suggested that blocking the growth of the visceral adipose tissues by angiogenic inhibitors can reduce the progress of NAFLD [66]. Furthermore, Li-Ying et al. have reported that VEGF is significantly upregulated at the RNA level in NAFLD [67], and the high concentration of VEGF was associated with more severe forms of NAFLD [68].

The findings of the current study are in agreement with the hypothesis of both of Gao et al. and Kim et al. It was revealed that 50% ME of *P. niruri* was effective in attenuating NAFLD, preventing fibrosis, and controlling the levels of serum adipocytokines, and exhibited, at the same time, a potent antiangiogenic activity. The anti-fibrotic activity of *P. niruri* was associated with significant antiangiogenic effect by preventing the development of rat aorta micro vessels, diminishing the migration and differentiation of endothelial cells.

Our current data about the inhibitory effect of *P. niruri* of VEGF are in accordance with the previous report which suggest that blocking VEGF, the activator of HSC, is one of the mechanisms of inhibiting fibrosis [69].

Furthermore, the results of MTT test with rat aortic ring analyse and the effect on EA.hy926 cells confirmed that the antiangiogenic potency verified by *P. niruri* is not related to the cytotoxic nature, but is possibly more related to blocking specific angiogenesis cascades.

Standardization of current 50% *P. niruri* extract revealed that ellagic acid and phyllanthin were major components [14]. Therefore, its anti-fibrotic effects are mostly owed to ellagic acid, which has high affinity for PPARα, and can enhance fatty acid oxidation by transactivation of the PPARα pathway. Ellagic acid can actively stimulate mitochondrial β oxidation of FFAs by promoting the activity of carnitine palmitoyltransferase 1 [70]. Ellagic acid also possesses a remarkable hepatoprotective activity, which is attributable to its role in preventing lipid peroxidation and enhancing hepatic defenses against oxidative stress [71]. In addition, we propose that the ellagic acid content in our extract was the reason for enhancing insulin signaling by stimulating adiponectin activity. Such an effect of ellagic acid was confirmed by a recent study [72]. Our results are also in agreement with an earlier report by Panchal, Ward [73] about the role of ellagic acid in attenuating the onset of diet-induced metabolic syndrome in rats.

Phyllanthin, the other major component of the current extract, has been shown to be capable of reducing the expression of lipolytic genes in white adipose tissues of mice, and can reverse oxidative stress and inflammation in the liver and adipose tissues. It precipitates a significant reduction of liver lipids, serum triglycerides, and free fatty acids in HFD-fed mice. It was also shown to be active in terms of decreasing serum pro-inflammatory cytokines and insulin resistance [74]. More importantly, phyllanthin acted as an essential anti-fibrotic agent in our extract by preventing the activation of nuclear factor-kB [75].

In conclusion, our results clearly demonstrated that eight weeks of HFD diet feeding in SD rats induced a significant dysfunction in adipose tissues among NAFLD rats, shown by the major reduction in adiponectin, and the considerable increase in vaspin, progranulin, RBP4, IL-6, and TNFα, leading to upregulation of PPARγ, SLC10A2, and Coll α1 genes, which are all involved in insulin resistance, hepatic steatosis, and fibrosis. Our data also revealed that oral administration of 1000 mg/kg of 50% ME of *P. niruri* extract and 500 mg/kg of metformin prevented hepatic fibrosis. *P. niruri* extract exhibited a comparable effect as metformin, indicating that it can be a naturally effective therapeutic choice. We also propose that the mechanism by which *P. niruri* elucidated this activity is multifactorial (Figure 9), but it is mainly based on decreasing the systemic insulin resistance in both adipose tissues and liver. With *P. niruri* treatment, the abnormal alterations in all adipocytokines were rescued, and led to consequent similar improvements in insulin signaling inside the liver. This improvement resulted in a reduction of inflammation, as well as fat accumulation, and contributed in the downregulation of PPARγ, SLC10A2, and Coll α1 genes. Moreover, the beneficial effects of 50% ME of *P. niruri* against NAFLD progression may be also due to its potent antiangiogenic effect, which is responsible for attenuating the pathological expansion of adipose tissues and regulating its function, leading to the adjustment of adipocytokine secretion, improvement in insulin signaling, and prevention of inflammation and fibrosis in the liver. However, the limitation of the current study is that further toxicity studies of 50% ME of *P. niruri* are still needed, and additional studies are required to elucidate the beneficial effect of formulating *P. niruri* with other herbal extracts to integrate the efficacy against NAFLD.

## Figures and Tables

**Table 1:** Specifications and details of target and reference genes.

Gene Symbol	Gen Name	Taqman Gene Expression Assay Number	Genbank Accession Number	Amplicon Length
PPARγ	Peroxisomal proliferator-activated receptor-γ	Rn00440945_m1	NM_001145366.1	105
SLC10A2	Solute carrier family 10 (sodium/bile acid cotransporter), member 2	Rn01414698_m1	NM_001270862.1	102
CFD	Complement factor D (adipsin)	Rn01535436_g1	NM_001077642.1	65
PNPLA2	Patatin-like phospholipase domain containing 2	Rn01479969_m1	NM_001108509.2	115
COL1A1	Collagen, type I, alpha 1	Rn01463848_m1	NM_053304.1	115
HPRT-1	Hypoxanthine phosphoribosyltransferase 1 (reference gene)	Rn01527840_m1	NM_012583.2	64
GAPDH	Glyceraldehyde-3-phosphate dehydrogenase (reference gene)	Rn0177563_g1	NM_017008.4	174

**Table 2:** The effects of 50% ME of *P. niruri* extract towards different parameters of tube formation.

Treatment	Covered Area [%]	Total Tube Length [px]	Total Branching Points	Total Loops	Inhibition%
**Negative control**	61.08 ± 2.43	12,789.80 ± 1069	59.20 ± 9.60	16.60 ± 3.11	----
**Positive control (betulinic acid)**	14.45 ± 0.98 ^b^	4107.60 ± 723.53 ^b^	14.80 ± 3.15 ^b^	0.60 ± 0.60 ^b^	67.88
**Extract IC_25_**	38.78 ±3.19 ^b^	9736.40 ±292.9 ^a^	38.80 ± 1.460 ^a^	6.20 ± 2.03 ^b^	31.94
**Extract IC_50_**	17.50 ± 0.82 ^b^	7566.80 ± 302.24 ^b^	29.00 ± 2.35 ^b^	2.00 ± 0.55 ^b^	40.84
**Extract IC_90_**	7.56 ± 0.36 ^b^	4128.60 ± 119.72 ^b^	2.40 ± 0.81 ^b^	0.00 ± 0.00 ^b^	67.72

^a^ *p* ≤ 0.05 and ^b^ *p* ≤ 0.01 vs. negative control, IC_25_ = 68 µg/mL, IC_50_ 136 µg/mL, and IC_90_ = 244 µg/mL, and betulinic acid = 10 µg/mL.
